# Soybean vs. Pea Bean in the Diet of Medium-Growing Broiler Chickens Raised under Semi-Intensive Conditions of Inner Mediterranean Areas: Growth Performance and Environmental Impact

**DOI:** 10.3390/ani12050649

**Published:** 2022-03-03

**Authors:** Antonella Fatica, Francesco Fantuz, Mengjun Wu, Siria Tavaniello, Giuseppe Maiorano, Elisabetta Salimei

**Affiliations:** 1 Dipartimento Agricoltura, Ambiente, Alimenti, Università degli Studi del Molise, Via Francesco de Sanctis 1, 86100 Campobasso, Italy; m.wu@studenti.unimol.it (M.W.); siria.tavaniello@unimol.it (S.T.); maior@unimol.it (G.M.); salimei@unimol.it (E.S.); 2 Scuola di Bioscienze e Medicina Veterinaria, Università degli Studi di Camerino, Via Gentile III Da Varano, 62032 Camerino, Italy; francesco.fantuz@unicam.it

**Keywords:** broiler chickens, diet, genotype, protein sources, growth, economic cost, environment, greenhouse gas, GLEAM-i

## Abstract

**Simple Summary:**

Growth performance of medium-growing broiler chickens raised under semi-intensive conditions was evaluated comparing two different dietary treatments and two different genotypes. Starting from the dietary ingredients traditionally used, soybean was totally replaced by pea bean. The experimental diets were also compared from an environmental point of view simulating the annual global warming potential of the diets. Neither genotype nor dietary treatment affected feed consumption and growth performance, suggesting that the total replacement of soybean with pea bean can be a valuable feeding strategy, also from the economic point of view. A diet containing pea slightly reduced the total greenhouse gas and carbon dioxide emitted on a yearly basis. The results of the present study suggest that replacing soybean with pea bean can be a sustainable feeding strategy in broiler chicken production of inner Mediterranean areas, from both economic and environmental perspectives.

**Abstract:**

The effects of *Glycine max* L. (SOY) vs. *Pisum sativum* L. (PEA) in the diet were investigated on in vivo performance of two medium-growing broiler genotypes and on environmental impact. Sixty Kabir Red Plus and sixty New Red chickens were randomly allocated in 20 pens (*n* = 6 birds per pen). Each pen, i.e., experimental unit, received 1.18 kg dry matter (DM) including soybean (3.39%) in SOY, or pea bean (6.78%) in PEA groups. DM intake, DM refusals and bodyweight (BW) were recorded on pen basis, and average daily gain (ADG) and feed conversion rate (FCR) were calculated. Data on in vivo performance were processed by ANOVA General Linear Model followed by Student–Newman–Keuls post hoc test. Greenhouse gas (GHG) emissions were evaluated on a year basis by GLEAM-i software. The diet did not affect (*p* > 0.05) DM intake and DM refusals, BW, ADG and FCR. Diet x genotypes affected (*p* < 0.05) DM intake and DM refusals. PEA diet, cheaper than SOY diet, allowed the reduction in total GHG and CO_2_, and of meat emission intensity. However, an increase in total CH_4_ and N_2_O emissions was observed. The replacement of soybean with pea bean can represent a possible management strategy to reach trade-off between good farming practices and environmental protection on small-scale poultry farms of inner Mediterranean areas.

## 1. Introduction

Soybean (*Glycine max* L.), the most common protein source in the poultry diet, is mainly imported from non-European countries [1,2]. Due to the increased soybean price, ranging from 410 to 710 EUR/t in the last three years [3], the feed costs increased up to 70% of the broiler chicken production total expenses [4]. For these reasons, alternative protein and energy sources and agroindustry by-products locally available have been increasingly considered as cheaper choice for poultry dietary vegetal ingredients [5]. Among them, pea bean (*Pisum sativum* L.) is a legume crop used in human and animal nutrition characterized by good nutritive value, high starch content with a reported low susceptibility to hydrolytic enzymes, and by the possible presence of antinutritional factors, i.e., condensed tannins and protease inhibitors [2,6]. Moreover, because consumers increasingly consider ethical issues related to meat production besides safety and quality, the inclusion of pea bean or other legumes, i.e., faba bean (*Vicia faba* L.) and lupin seeds (*Lupinus albus* L.), appears more widespread in broiler diets in semi-intensive rearing systems [4].

Diet, together with genotype, age and sex of birds, and their management systems, is an important factor influencing animal growth and quality of production. Furthermore, focusing on well-being and housing conditions, chicken meat production is more oriented towards less intensive systems, for which medium- and slow-growing broiler strains have been recommended [7]. In fact, medium- and slow-growing chickens are more suitable for alternative production systems for their higher vitality, disease resistance, and adaptability to outdoor conditions than fast-growing chickens [8,9,10,11].

The increasing concern on agriculture sustainability and food quality and safety are driving consumers to prefer short animal production chains perceived as natural, respectful of animal welfare, and environmentally friendly [12]. Accordingly, poultry meat produced in outdoor or semi-intensive rearing systems could represent a valid market choice [13], especially for small-scale farms of inner Mediterranean areas. There is also a tendency towards renewable resources in agriculture, facing the challenge of producing foods and feeds with lower environmental impact [14]. For the 2010 reference period, greenhouse gas (GHG) emissions from livestock supply chains were estimated at 8.1 Gt CO_2_-equivalent/year [15]; consequently, the improved global sustainability of feed production processes encouraged the poultry sector to limit its environmental load, expressed in terms of GHG [16]. Environmental impacts associated with broiler production are derived mainly from the dietary raw materials, so that it is increasingly important to pay attention to diet formulation, besides energy use and efficiency, growth rate of the selected genotypes, and animal and manure management practices [16]. Carbon footprint is a measure of environmental sustainability, referring to the total amount of GHG emissions produced during the entire life cycle of a specific product or service, that contributes to the global warming potential (GWP) and to the climate change within a 100-year period [17]. Considering the entire life cycle of a product, i.e., from cradle to grave, life cycle assessment (LCA) represents an internationally standardized method (ISO 14040:2006) that quantifies the environmental pressure related to the production of goods and commodities, and its use can result in an improved production process as a strategic management and decision-making tool in the frame of a more sustainable and efficient society [14]. The GLEAM-i model, based on both LCA and geographic information system (GIS) methodologies, was developed by FAO to estimate the annual environmental impacts of several livestock productions from different regions of the world [18].

The present research aimed to assess the effects of two different dietary protein sources (soybean vs. pea bean) on growth performance of two different chicken genotypes (Kabir Red Plus vs. New Red) raised according to the traditional schemes of semi-intensive system in inner areas of central-south Italy. Besides growth performance, the study was extended to the environmental impact of the dietary treatments.

## 2. Materials and Methods

### 2.1. Ethics

The on-field trial was performed as a routine production cycle in small-scale farming conditions, in accordance with the Italian law on protection of birds raised for meat production (D. Lgs. 181/2010). The ethical approval of the research protocol was waived in accordance with the European Commission legislation (Dir. No. 2010/63/EU).

### 2.2. Animals, Diets, and Experimental Design

The on-field trial was carried out in a private poultry farm located in Benevento municipality (41°07′12.8″ N 14°43′34.8″ E, Italy), in a hilly area at an altitude of about 300 m. The experiment was conducted from October to December 2019, under semi-intensive rearing conditions. A total of 120 medium-growing male chickens, Kabir Red Plus (K, *n* = 60) and New Red (NR, *n* = 60) were used.

After hatching, all the chicks were vaccinated according to the current commercial practice (Infectious Bronchitis Virus, Marek’s disease virus, Newcastle and Gumboro disease). Chickens were housed at 20 days of age and randomly allotted according to genotype (K and NR) and diet (soybean, SOY and pea bean, PEA), in similar spatial and environmental conditions. There were 5 replicates with 6 birds/pen for each group (K-SOY, NR-SOY, K-PEA, NR-PEA) homogeneous for genotype, diet, and initial body weight. Pens were made by wire mesh screen on four sides and dirt floor where straw was added weekly to create a permanent litter on which manure was removed at the end of the trial. Each pen (1.60 m × 0.80 m × 1.50 m), representative of the experimental unit, was equipped with a single feeder and bottle-drinker to ensure free and simultaneous access to both feed and water. Pens were positioned under a covered structure characterized by two open sides, where environmental conditions, such as temperature, humidity, and lightness, were not controlled. Data about thermo-pluviometric conditions, temperature (°C), rainfalls (mm), and humidity (%), were collected from the weather station close to the selected farm [19].

The feeding trial lasted 36 days (from 47 to 83 days of age) preceded by an adaptation period of 45 days, as follows: for the first 15 d, all animals were fed ad libitum a commercial complete starter mixed feed (24% CP, 16.33 MJ/kg dry matter (DM) of apparent metabolizable energy, aME [20]) including coccidiostat additive. After that, the gradual adaptation of the animals to the adult experimental diets was performed in two phases. In the first one, which lasted 6 days, 15% adult diet and 85% starter diet was administered the first 3 days, and 30% adult diet and 70% starter diet was offered the last 3 days, while in the second phase, which lasted 4 days, 60% adult diet and 40% starter diet was distributed.

Two isonitrogenous and isoenergetic experimental diets were formulated according to birds’ nutritional needs [20], including either flaked soybean (SOY) or pea bean (PEA). Both diets also included faba bean (*Vicia faba* L.), as common protein source, and wheat products as dietary ingredients locally available (Table 1).

The chemical components reported in Table 1 were analysed according to the official methods [21] except for crude fibre, lysine, methionine and energy content, calculated from tabulated values for raw materials [22]. Each experimental unit (pen) received daily 1.18 kg DM mixed feed, grinded by a mechanical mill (1 mm). Diets were administered after the addition of water (1 kg dry feed: 2 L water) into two daily meals, in the morning (08:00) and in the afternoon (15:00).

### 2.3. Measurements and Recordings

Before each meal, mixed feeds administered to each experimental unit were weighed, and refusals were collected from the feeder of each pen at the end of the day. Every ten days, residuals were sampled and analysed for DM content, according to official method [21]. Daily DM intake was calculated as the difference between feedstuffs offered (kg DM per pen) and refusals (kg DM per pen).

Economic cost analysis of experimental diets were carried out according to Bologna Exchange Commodity weekly reports [3]. Despite the feeding trial lasted 36 days, dietary ingredient prices were collected and analysed as relating to the last three months of 2019, in order to consider the fluctuations of markets over a short–medium period, as suggested by Taylor [23].

Chickens were weighed on a pen basis at the beginning (0 day) and at 14, 28 and 36 days of the feeding trial. The average individual initial (0 day) body weight (BW) was: 1.45 (±0.19 SD) kg, 1.49 (±0.38) kg, 1.75 (±0.48) kg, 1.74 (±0.38) kg for K-SOY, K-PEA, NR-SOY, and NR-PEA, respectively. BW was recorded, and average daily gain (ADG) was determined. FCR was calculated as the ratio between DM intake and its relative ADG for the overall period. Dead chickens were recorded daily during the entire experimental period.

### 2.4. Environmental Impact

The carbon footprint of the reported case study was assessed by the Global Livestock Environmental Assessment Model—Interactive software GLEAM-i version 1.8 (Food Agriculture Organization of the United Nations, Rome, Italy) [18]. The emission intensity produced by the functional unit per kg of product was expressed as carbon dioxide equivalent emitted per kg of protein, i.e., kg CO_2_-eq/kg Prot [18].

To estimate the environmental impacts of the two investigated diets, SOY diet was defined as baseline case and PEA diet was defined as scenario case, according to GLEAM-i guidelines. The on-field trial data on feed ingredients (Table 1) were entered in the feed module of GLEAM-i, while input data for herd and manure modules are summarised in Table 2.

It is worth noting that the analysis process underwent some adaptations in feed data input due to software restrictions, i.e., “grains from wheat” was referred only to *Triticum aestivum* L., and legume bean sources were not differentiated except for soy, reported separately. To overcome these limits, the dietary wheat inclusion was expressed as *Triticum aestivum* L. instead of *Triticum durum* Desf., and both pea bean and faba bean ingredients were reported as “leguminous beans source”, by adding up the two protein source percentages.

### 2.5. Statistical Analyses

Data about daily DM intake and DM refusals were processed by analyses of variance followed by Student–Newman–Keuls (SNK) post hoc test. Data on BW, ADG and FCR were processed by analyses of variance according to general linear model (GLM) procedure using a full factorial design, including dietary treatment, genotype, and their interaction. The covariate effect of “body weight at d 0” was also included in both analyses. Results were presented as mean and standard error (SEM) unless otherwise stated, and differences were considered significant for *p* < 0.05. Statistical analyses were conducted by IBM SPSS Statistic Data Editor version 25 (Chicago, IL, USA).

## 3. Results

### 3.1. Climatic Characterization of Study Area

**Figure 1:**
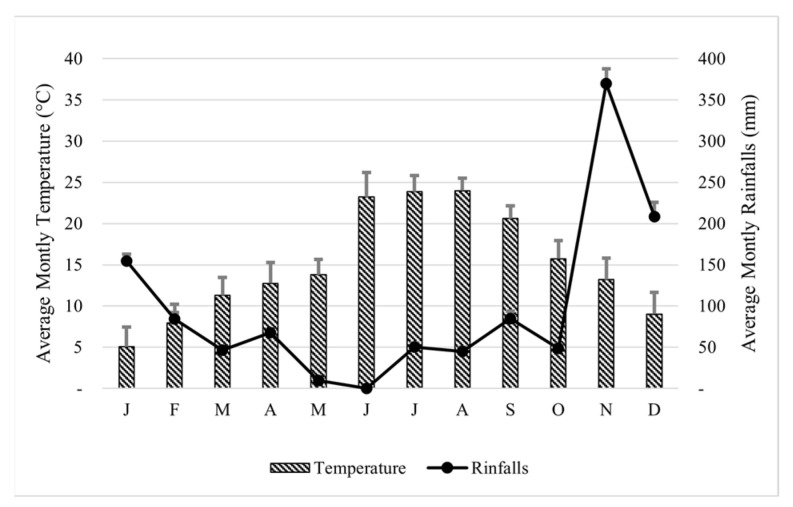
Monthly climatic characterization of studied area during 2019.

To define the climatic characterization of the farm area, Figure 1 shows the 2019 monthly values of temperature (°C) and rainfalls (mm). During the experimental period (October–December), temperature gradually decreased from 15.8 °C to 9.0 °C (Figure 1). The average rainfalls ranged from the minimum value in October (48.4 mm of rain) to the maximum value in November (369.6 mm of rain), while in December, precipitation averaged 208.2 mm (Figure 1). During the experiment, relative humidity averaged 80.4%, and the medium photoperiod was around 10 h light/14 h dark.

### 3.2. Dietary Treatment and Genotype: Effects on Feed Consumption

Neither the daily DM intake nor daily DM refusals were affected (*p* > 0.05) by dietary treatment and genotype (Table 3).

**Figure 2:**
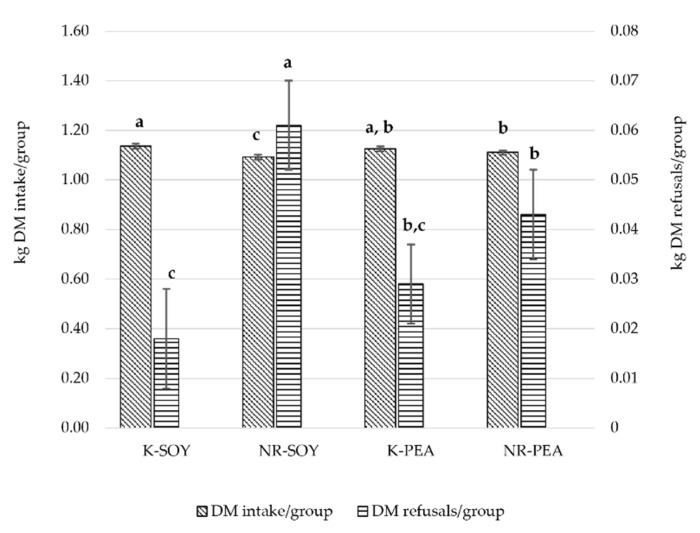
Daily group DM intake and DM refusals. ^a,b,c^ Mean values with different letters are significantly different (*p* < 0.05) within each variable.

The interaction diet × genotype was significant (*p* < 0.05; Table 3), and the effects of diet and genotype on group daily DM intake and DM refusals are displayed in Figure 2. NR chickens fed SOY diet showed lower DM intake and higher DM refusals compared to NR chickens fed PEA diet. Conversely, K chickens showed similar DM intake and DM refusals in both diets.

The calculated average individual DM intake (±SD) at the end of the feeding trial (83 days of age) was 0.189 (±0.004) kg, 0.178 (±0.005) kg, 0.169 (±0.007) kg, 0.184 (±0.005) kg for K-SOY, K-PEA, NR-SOY, and NR-PEA, respectively.

From an economical point of view, PEA diet was cheaper than SOY diet (−0.40% as fed and −5.00% as DM; Table 4). Changes in raw material prices for the last trimester of 2019, 2020 and 2021 are reported as supplementary material (Table S1).

### 3.3. Dietary Treatment and Genotype: Effects on Growth Performance

The effects of different dietary protein sources and genotype on productive performance of broiler chickens are reported in Table 5. The dietary treatment and genotype did not affect (*p* > 0.05) poultry BW (14, 28 and 36 days), and overall ADG and FCR (Table 5). No significant (*p* > 0.05) interaction diet × genotype was observed.

The calculated average individual body weight (±SD) at the end of feeding trial (83 days of age) was 2.47 (±0.05) kg, 2.40 (±0.03) kg, 2.83 (±0.06) kg, 2.86 (±0.06) kg for K-SOY, K-PEA, NR-SOY, and NR-PEA, respectively. Individual average daily gain (±SD) at the end of feeding trial (83 days of age) was calculated 27.0 (±1.2) g, 25.0 (±0.9) g, 30.0 (±1.4) g, 30.0 (±1.4) g for K-SOY, K-PEA, NR-SOY, and NR-PEA, respectively.

The observed mortality rate was 1.70%, as at 13 days and 20 days of feeding trial, two chickens (1 in K-SOY and 1 in NR-PEA pen) died due to unknown causes.

### 3.4. GLEAM-i Elaboration

Considering the LCA framework, i.e., from crop to farm, results on environmental impacts for SOY and PEA diet and variations (+/−delta, %), calculated on a year basis, are presented in Table 6. Total GHG emissions, expressed as kg CO_2_-eq/year, resulted higher in SOY than in PEA diet. More in detail, PEA diet allowed for the reduction in total GHG (−8.21%), total annual CO_2_ (kg CO_2_/year, −66.1%) and meat emission intensity (kg CO_2_-eq/kg Prot, −8.21%) (Table 6). However, an increase in total emissions of CH_4_ (kg CH_4_/year, +1.81%) and N_2_O (kg N_2_O/year, +9.00%) was observed, consistent with the increase in total DM consumption (+1.32%, *p* > 0.05; Table 3).

## 4. Discussion

Several factors affect poultry growth performance, i.e., age, sex, diet, genotype, housing system, stocking density and environmental factors. Among these, nutritional aspects are more relevant in growing birds; in fact, any change in nutrition is reported to suddenly reflect in chicken performance, due to peculiar physiology and gastrointestinal conformation [20,24,25]. The calculated average values of daily individual DM intake observed at 83 days of age were approximately twofold higher than those reported for Ross 308 chickens (42 days of age) fed raw field peas in partial replacement of soybean meal and corn [2]. A similar result was found in slow-growing chickens (61–80 days of age) fed faba bean in partial substitution of soybean [26]. In addition, the latter authors found lower daily weight gain and feed efficiency in birds fed faba bean compared to control ones until 60 days of age, while they did not find significant differences up to 120 days. The authors stated that the lower performance could be mainly attributed to raw faba bean in the starter diet, which is characterized by both low essential amino acid (methionine, cysteine, threonine, tryptophan) contents and the presence of several anti-nutritional factors. Generally, in intensive rearing systems, fast-growing chickens consume higher amounts of DM at the same age than slow-growing broilers, which is considered less efficient and sustainable [8]. The average values of feed consumption reported for slow-growing chickens, raised with outdoor access and fed a low nutrient diet until 13 weeks of age, resulted in slightly more than half of the values calculated in this study [27]. Similar average daily feed intake was reported also for slow-growing broilers (49 days of age) fed a diet containing high-protein micronized peas as a substitute of soybean meal [1]. Furthermore, average values of daily DM intake reported in literature for different genotypes and rearing systems are 0.09 kg DM/chicken at 42 days of age, 0.08 kg DM/chicken at 84 days of age, and 0.15 kg DM/chicken at 81 days of age [28,29,30].

The final BW of poultry is also influenced by the rearing system [12]. At the same age, chickens reared outdoor have a lower body weight than animals raised indoor, because of variations in temperature and physical exercise done by the animals, resulting in increased energy expenditure and higher feed conversion rate [1,4,31]. Furthermore, a negative correlation between birds’ BW and active behaviours is also reported [30]. Regarding the physical activity, under the same farm and feeding conditions, medium- and slow-growing birds have a more active behaviour and higher adaptability to the natural environment compared to fast-growing genotypes [30,32]. This can explain the observed BW values being lower than those reported by commercial information on the selected genotypes [33]. As a further confirmation, experimental groups were characterized by an average BW (83 days of age) close to that reported at 63 d of age for the same genotypes raised under intensive conditions [33]. The final BW of K-SOY and K-PEA chickens from the current study were close to the final BW reported for 42 days old broiler chickens fed raw field pea [2], and to values reported for fast-growing broilers (42 days of age) fed diets containing olive pulp [34]. Differently, a higher final BW was observed for Kabir chickens reared in spring under organic farming system with outdoor access [35]. The final BW of NR-SOY and NR-PEA groups was higher than fast-growing broilers fed a diet containing micronized and dehulled peas [1], or faba, pea, lupin beans, or soybean meal [36].

The ADG values observed in PEA and SOY groups were lower than those reported for fast-growing chickens raised for 56 days in a semi-intensive system, but were similar to the ADG reported for broilers of 61–80 days old fed soybean diet [12,26]. The observed results on ADG could indicate higher adaptability of K chickens to the environmental and management conditions of the current study. Results about FCR were higher than those observed for broilers fed soybean diet (61 and 80 days of age), and for 42 days old broiler chickens fed a diet with moderate inclusion of field peas [2,26]. The inclusion of a high percentage of wheat by-products explains the observed low feed efficiency, as FCR is a function of animal genetics and age, quality of dietary ingredients and environmental conditions [20,28,37]. In this regard, it is worth noting that the high fibre content of the experimental diets was higher than diets usually implemented in intensive broiler farming systems.

The mortality rate observed in the present study (1.70%) was higher than the range 0.60–1.30% reported for 84 days old dual purpose chickens [29], but consistent with that reported for crossbred chickens at 91 days of age [38].

From the economic point of view PEA diet resulted cheaper than SOY diet; however, changes of ingredient prices could be attributable to a disequilibrium between demand and supply of raw materials [39]. In the last two years, the increasing trend of raw material prices has been affected not only by the global reduction in ingredients availability, but also by a speculative factor possibly driven by an increased worldwide demand for cereals and oil seeds, following the deep crisis due to both COVID-19 pandemic and African swine fever outbreak [39].

Referring to the environmental impacts, the most common greenhouse gases emitted in the atmosphere from the poultry sector, which negatively affect animals and workers, are CO_2_, N_2_O, and CH_4_, closely related to dietary components and energy content, and to the energy input needed to produce mineral fertilizers, essential for crop intensive production systems [40]. CO_2_ is largely produced by burning biomass and fossil fuels (i.e., coal, oil, and gas) during the fertilizer manufacturing, and as a consequence of land use changes and industrial processes [41]. CH_4_ is the major component of natural gases whose production is associated with animal husbandry, being the emissions most closely related to the diet composition and its digestibility, besides manure management [41]. Agriculture activities, such as soil and manure management, sewage treatment, chemical industrial processes, and fossil fuel combustion, are the main sources of N_2_O [41]. In the poultry sector, the production of N_2_O is primarily associated with the high nutrient-feed requirements, but it is also linked to the emissions from arable lands and fertilizers production as well as it being produced by nitrification and denitrification processes [42,43]. The different gas emissions from SOY and PEA diets could be attributed to cultivation area, cultivation technique, as well as transportation and processing of raw materials. It is worth noting that GLEAM-i benchmark data (default) for the Italian poultry population estimate a meat production around 745 Gt meat/year, while SOY and PEA diet data provided only 87 kg meat/year (Table 6). Consequently, the total GHG emission linked to the meat production for the Italian poultry sector is estimated at 1415 Gt CO_2_-eq/year, while approximately 550 kg CO_2_-eq/year was the emission evaluated for both diets in the present case study. However, the meat emission intensity, reported equal to 13.3 kg CO_2_-eq/kg Prot for the default data, was about three times higher for SOY and PEA diet (46.8 CO_2_-eq/kg Prot vs. 42.9 CO_2_-eq/kg Prot, respectively), likely related to farming system and growth rate of the current case study. Default data are indeed based on intensive rearing systems with controlled conditions for meat production and fast-growing genotypes, according to FAO guidelines [44]. Conversely, birds in the present study were reared under semi-intensive conditions, in winter, with no controlled environmental conditions, and fed diets mainly containing ingredients available locally. The meat emission intensity (CO_2_-eq/kg protein) of the two dietary treatments (SOY vs. PEA) was very close (Table 6) and consistent with the average live weight (2.62 kg/head for SOY vs. 2.57 kg/head for PEA diet). The observed data, 6.64 kg CO_2_/kg BW (SOY) vs. 6.10 kg CO_2_/kg BW (PEA), were higher than the GWP derived from feed and manure modules for small-scale broiler (five annual cycles), where the emission of 3.68 kg CO_2_-eq was estimated for an average carcass weight of 1.20 kg [45]. Furthermore, the environmental impact from the PEA diet was slightly above 5.97 kg CO_2_-eq/kg carcass weight reported by Espino and Bellotindos [45]. Moreover, according to Zervas and Tsiplakou [46], for chickens raised under conventional intensive system, the global warming potential ranged from 1.40 CO_2_-eq/kg BW to 2.30 CO_2_-eq/kg BW. It must be considered in this regard that each productive system is characterized not only by the specific management and feeding organization, but also by the quality of the final products.

## 5. Conclusions

Dietary treatment as well as chicken genotype did not significantly affect dry matter intake and growing performance of broilers raised under semi-intensive conditions, although a significant interaction between diet and genotype was observed. A diet including pea bean was cheaper than that including soybean in the investigated short–medium period. From an environmental point of view, as the impact intensity is strongly connected to the feed production practices and diet ingredients, a PEA diet slightly reduced the total annual GHG emissions and those linked to meat production, as well as the CO_2_ emissions. However, the introduction in the diet of pea bean resulted in an increased annual emission of N_2_O and CH_4_.

The results of the present study suggest that replacing soybean with pea bean can be a sustainable feeding strategy from both economic and environmental perspectives, as further added value to local small-scale poultry production of inner Mediterranean areas.

## Figures and Tables

**Table 1:** Ingredients, chemical composition and energy content of SOY and PEA experimental diets daily administered to broiler chickens.

Feed Administered	SOY	PEA
Ingredients, g/100 g DM		
Wheat bran	49.15	47.46
Durum wheat	25.42	26.27
Corn meal	15.26	12.71
Faba bean	6.78	6.78
Pea bean	-	6.78
Soybean flaked, 37% CP	3.39	-
Total	100	100
Analysed results		
DM, g/kg DM	889.0	892.0
CP, g/kg DM	186.0	186.0
EE, g/kg DM	48.2	44.5
Ash, g/kg DM	49.7	48.3
Calculated analysis		
CF, g/kg DM	81.6	81.4
Lys, g/kg DM	6.70	6.90
Met, g/kg DM	2.50	2.40
aME, MJ/kg DM	13.3	13.2

SOY = SOY diet; PEA = PEA diet; DM = dry matter; CP = crude protein; EE = ether extract; CF = crude fibre; Lys = lysine; Met = methionine; aME = apparent metabolizable energy.

**Table 2:** GLEAM-i input for SOY and PEA diets.

Parameter *	Unit	SOY (Baseline)	PEA (Scenario)
Herd module			
Number of animals	n	60	60
Live weight at slaughter	kg	2.62	2.57
Death rate of adult broilers	%	1.70	1.70
Manure module			
Poultry manure with litter	%	100	100

* See Table 1 for feed ingredients, i.e., feed module. SOY (baseline) = SOY diet; PEA (scenario) = PEA diet.

**Table 3:** Effect of dietary treatment, genotype, and their interaction on daily DM intake and DM refusals per pen.

	Diet		SEM	Genotype		SEM	*p*-Value		
	SOY	PEA		KABIR	NEW RED		D	G	D × G
DM intake, kg	1.11	1.12	0.04	1.13	1.10	0.009	ns	ns	*
DM refusals, kg	0.04	0.04	0.04	0.02	0.05	0.008	ns	ns	*

DM = dry matter; D = diet; G = genotype; SEM = standard error of mean; * *p* < 0.05; ns = not significant.

**Table 4:** Raw materials price average values (±SD) and calculated feeding costs for each experimental treatment for the last trimester of 2019.

Feed Ingredients	Price ^a^ (EUR/t)	Diet Cost (EUR/100 kg as Fed)		Diet Cost (EUR/kg DM)	
		SOY	PEA	SOY	PEA
Wheat bran	161.6 (±13.5)	8.42	8.08	0.08	0.08
Durum Wheat	266.7 (±8.31)	6.40	6.67	0.07	0.07
Corn meal	174.8 (±0.39)	2.56	2.18	0.03	0.02
Faba bean	278.6 (±1.92)	1.74	1.74	0.02	0.2
Pea bean	234.4 (±8.41)	-	1.46	-	0.2
Soybean flaked, 37% CP	367.5 (±3.78)	1.10	-	0.01	-
Total cost		20.22	20.14	0.21	0.20
Variation ^b^, %			−0.40		−5.00

^a^ Based on the average price of the last trimester of 2019 from Bologna Exchange Commodity [3]. ^b^ Variation = ((PEA-SOY)/PEA) ∗ 100. DM = dry matter; CP = crude protein.

**Table 5:** Effect of dietary protein sources, genotype, and their interaction on growth performance of broiler chickens per pen.

	Diet		SEM	Genotype		SEM	*p*-Value		
	SOY	PEA		KABIR	NEW RED		D	G	D × G
BW, kg/group									
14 day	12.8	12.5	0.11	14.4	12.9	0.23	ns	ns	ns
28 day	15.6	15.2	0.37	15.6	15.2	0.74	ns	ns	ns
36 day	15.7	15.4	0.40	16.2	15.0	0.82	ns	ns	ns
ADG, kg/day	0.17	0.16	0.01	0.18	0.15	0.02	ns	ns	ns
FCR, kg DM intake/kg gain	6.95	7.25	0.56	6.40	7.76	1.13	ns	ns	ns

BW = body weight; ADG = average daily gain; FCR = feed conversion rate; SEM = standard error of mean; D = diet, G = genotype; ns = not significant.

**Table 6:** Environmental impacts of selected diets ^a^.

Parameters	Unit	SOY (Baseline)	PEA (Scenario)	Delta (%)
Total GHG emissions	kg CO_2_-eq/year	583.3	535.3	−8.21
Total CO_2_	kg CO_2_/year	125.9	42.7	−66.1
Total CH_4_	kg CH_4_/year	2.42	2.46	+1.81
Total N_2_O	kg N_2_O/year	1.26	1.37	+9.00
Total feed intake	kg DM/year	2261.2	2901.0	+1.32
System meat production	kg/year	87.8	87.8	+0.00
GHG emissions linked to meat production	kg CO_2_/year	583.3	535.3	−8.21
Meat emission intensity	kg CO_2_-eq/kg Prot	46.6	42.8	−8.21

^a^ Calculated for 60 birds/dietary treatment. SOY (baseline) = SOY diet; PEA (scenario) = PEA diet; DM = dry matter; GHG = greenhouse gas; Prot = protein. Delta = ((scenario-baseline)/baseline) ∗ 100.

## Data Availability

Data presented in this study are available on request from corresponding author.

## Supplementary Materials

The following are available online at https://www.mdpi.com/article/10.3390/ani12050649/s1, Table S1: Raw materials price average values (±SD) and calculated feeding costs for each experimental treatment for the last trimester of 2019, 2020, and 2021.
