# Supplementary material for: Soybean vs. Pea Bean in the Diet of Medium-Growing Broiler Chickens Raised under Semi-Intensive Conditions of Inner Mediterranean Areas: Growth Performance and Environmental Impact
Source: Animals (Basel). 2022 Mar 3;12(5):649. doi: 10.3390/ani12050649 (PMC8909337; doi:10.3390/ani12050649)
Supplement: Supplementary file 1 [file animals-12-00649-s001.zip › animals-1580613-supplementary.pdf]

Supplementary Material

Table S1. Raw materials price average values (± SD) and calculated feeding costs for each experimental treatment for the last trimester of 2019, 2020, and 2021.

Feed ingredients	Price [a] (€/t)	Diet cost (€/100 kg as fed)		Diet cost (€/kg DM)	
		SOY	PEA	SOY	PEA
**2019**					
Wheat bran	161.6 (± 13.5)	8.42	8.08	0.08	0.08
Durum Wheat	266.7 (± 8.31)	6.40	6.67	0.07	0.07
Corn meal	174.8 (± 0.39)	2.56	2.18	0.03	0.02
Faba bean	278.6 (± 1.92)	1.74	1.74	0.02	0.2
Pea bean	234.4 (± 8.41)	-	1.46	-	0.2
Soybean flaked, 37 % CP	367.5 (± 3.78)	1.10	-	0.01	-
**Total cost**		20.22	20.14	0.21	0.20
**Variation** [b], %			- 0.40		- 5.00
**2020**					
Wheat bran	197.9 (± 18.5)	10.31	9.89	0.10	0.09
Durum Wheat	297.8 (± 7.97)	7.15	7.45	0.08	0.08
Corn meal	195.3 (± 5.82)	2.86	2.44	0.03	0.03
Faba bean	289.0 (± 3.77)	1.81	1.81	0.02	0.02
Pea bean	250.7 (± 13.9)	-	1.57	-	0.02
Soybean flaked, 37 % CP	433.9 (± 20.1)	1.30	-	0.02	-
**Total cost**		23.43	23.16	0.24	0.23
**Variation** [b], %			- 1.17		- 4.35
**2021**					
Wheat bran	214.6 (± 22.9)	11.18	10.73	0.11	0.10
Durum Wheat	542.3 (± 6.93)	13.01	13.56	0.14	0.14
Corn meal	295.1 (± 5.94)	4.32	3.69	0.05	0.04
Faba bean	377.0 (± 16.9)	2.36	2.36	0.03	0.03
Pea bean	320.5 (± 0.00)	-	2.00	-	0.02
Soybean flaked, 37 % CP	528.1 (± 14.8)	1.58	-	0.02	-
**Total cost**		32.46	32.34	0.332	0.329
**Variation** [b], %			- 0.37		- 0.91

[a] Based on the average price of the last trimester of 2019, 2020, and 2021 from Bologna Exchange Commodity [3].
[b] Variation = ((PEA-SOY)/PEA) * 100.
DM = dry matter; CP = crude protein.
